# A Deep Prompt-Based Chain-of-Thought Approach to Harmful Euphemism Detection in Social Networks

**DOI:** 10.3390/e28050560

**Published:** 2026-05-17

**Authors:** Siyu Xie, Gang Zhou, Haizhou Wang

**Affiliations:** School of Cyber Science and Engineering, Sichuan University, Chengdu 610207, China; xiesiyu_more@163.com (S.X.); 18782726208@163.com (G.Z.)

**Keywords:** harmful euphemism, semantic perception, social network, deep learning

## Abstract

In recent years, cyberspace governance has become a critical component of national security strategies worldwide. Although social network platforms provide users with convenient channels for expression and information acquisition, unregulated, harmful euphemisms have become increasingly prevalent. These euphemisms disrupt the order of the digital space and trigger secondary harms such as cyberbullying and regional discrimination. Currently, researches on Chinese harmful euphemism detection face three key challenges: the lack of large-scale annotated datasets, the cognitive reasoning deficit in lightweight models, and the latency constraints of Large Language Models (LLMs), which collectively constrain detection performance and real-world generalization. To address these issues, this study first collected a large corpus from social networking platforms and constructed a fine-grained annotated harmful euphemism dataset. Then, a representation learning framework was designed by integrating deep prompt-based chain-of-thought reasoning with multi-head contrastive learning. This framework introduces external knowledge from LLMs to enhance the diversity and precision of semantic representations. Finally, a multi-dimensional semantic perception fusion framework was proposed. It incorporates multiple semantic perception channels and a cross-channel dynamic fusion mechanism, enabling the model to better capture implicit semantics and integrate external contextual knowledge. Experimental results show that our approach significantly outperforms state-of-the-art lightweight models. While large-scale LLMs exhibit superior zero-shot transferability in cross-domain tasks, our proposed model maintains highly competitive performance with substantially lower inference latency and computational overhead. This research provides a novel methodological and technical foundation for detecting harmful euphemisms in social networks.

## 1. Introduction

The rapid growth of social networking platforms has greatly facilitated information dissemination, but it has also led to a surge in malicious content, posing significant challenges to the online ecosystem [1]. Among various forms of harmful content, harmful euphemisms constitute a particularly elusive category, in which illegal, discriminatory, or violent intentions are conveyed covertly. Such expressions employ linguistic strategies—including homophones, abbreviations, metonymy, and metaphor—to superficially “sanitize” sensitive content and evade both automated moderation and manual review. Harmful euphemisms were initially prevalent in the dark web and underground forums—often referred to as “underground jargon” [2]—where they facilitated illicit activities such as drug trafficking, human exploitation, and the illegal arms trade. However, with the widespread adoption of social media and the increasing “gamification” of online discourse, these expressions have gradually permeated mainstream platforms. They have evolved into more subtle, dynamic, and socially embedded forms.

Against this backdrop, harmful euphemism detection has emerged as a critical research direction in cyberspace content security. Nevertheless, due to their cryptic semantics and rapid evolution, existing detection models struggle to effectively identify such implicit harmful content. These challenges can be summarized as follows:

1. Data Scarcity: There is a lack of large-scale, fine-grained datasets with contextual semantic annotations, which limits models’ semantic understanding and generalization capabilities.
2. Cognitive Reasoning Deficit in Lightweight Models: Existing methods largely rely on keywords or explicit features extracted by conventional pre-trained language models (PLMs). However, euphemistic expressions often depend heavily on contextual semantics and sociocultural backgrounds. Current lightweight models possess limited capacity for external knowledge integration and explicit logical deduction. This limitation significantly hinders detection accuracy and robustness when countering complex rhetorical disguises.
3. Latency Constraints of Large Language Models (LLMs): While modern LLMs possess strong zero-shot reasoning abilities capable of deciphering implicit intents, their large parameter sizes incur prohibitive computational overhead. This renders direct LLM deployment infeasible for the microsecond-level, high-concurrency real-time moderation demanded by modern social networks.

To address these issues, we propose a harmful euphemism detection model based on multi-dimensional semantic-aware fusion. The specific contributions of this work are as follows:

• We construct a large-scale Chinese harmful euphemism dataset with fine-grained semantic annotations. The dataset is built from the Bilibili platform and covers multiple dimensions, including topic categories, attack types, euphemistic expressions, and their corresponding explanations. To the best of our knowledge, this is the first dataset to incorporate explicit semantic explanations of harmful euphemisms.
• We propose a novel representation learning framework that integrates chain-of-thought (CoT) prompting with multi-head contrastive learning. By leveraging external knowledge from LLMs in an offline manner, the framework enhances the diversity and precision of semantic representations while avoiding the computational overhead of online inference.
• We develop a multi-dimensional semantic-aware detection model with dynamic fusion. The proposed architecture captures implicit semantics and contextual dependencies more effectively. It achieves strong performance while maintaining low inference latency.

## 2. Related Work

With the rapid growth of user bases and information flow on social networks, harmful euphemisms have increasingly exhibited greater concealment, morphological variation, and rapid diffusion across various platforms, which pose severe challenges to automated detection. The existing research has predominantly focused on explicit harmful speech [3], while less attention has been given to implicit and euphemistic harmful expressions disguised through homophones, metaphors, and encoding [4]. Early works mostly relied on keyword- or rule-based detection techniques, such as sensitive lexicons, blocklists, and allowlist/denylist mechanisms [5,6]. These methods struggle to model the deep semantics and contextual dependencies of complex and ever-changing euphemistic expressions, and thus exhibit clear limitations. Subsequent research has gradually evolved from simple vocabulary matching to more comprehensive methodological frameworks, oriented toward semantic representation learning, external knowledge modeling, and multimodal fusion. Table 1 summarizes key existing studies, comparing the datasets, backbone models, and their use of external knowledge. Overall, the existing research follows a clear evolutionary trajectory.

### 2.1. Semantics-Driven Representation of Harmful Euphemisms

To address the limitations of rule- and lexicon-based methods in handling novel and concealed euphemistic expressions, a line of research has shifted toward distributed semantic modeling. Early works mostly utilized static word embeddings such as Word2Vec to capture word co-occurrence patterns in corpora via distributed representations. Yuan et al. [7] developed neural models based on static word embeddings to analyze dark web illicit trade corpora, identifying euphemisms with significant semantic deviations and inferring their true meanings through semantic mapping. Taylor et al. [8] combined word embeddings with PageRank, using community detection methods to identify novel harmful euphemisms generated by extremist communities. Magu et al. [25] integrated word embeddings with network structure analysis to expand unknown euphemisms and related harmful texts based on known argots, demonstrating a hybrid semantic–graph-based detection paradigm. Targeting Chinese scenarios, Wang et al. [26] systematically evaluated various typical word embedding models, automatically discovering novel harmful euphemisms through semantic similarity measurement. They further introduced features such as pronunciation and glyphs, as well as domain-incremental training strategies.

With the development of deep learning and pre-trained language models, research has gradually shifted from static word embeddings to contextualized representations. Zhu et al. [9] first introduced BERT into the content moderation task, enabling the detection of euphemistic expressions in a self-supervised manner. Huang et al. [10] proposed DJPD, a low-frequency euphemism detection model, leveraging Transformer architectures to better model low-frequency and rare euphemistic expressions. Guan et al. [11] further constructed JargonFM, a multi-mode euphemism interpreter supporting category prediction, similar expression discovery, and representative text selection, thereby forming a unified framework for identifying and interpreting harmful euphemisms.

However, despite the continuous optimization of representation structures in the aforementioned models, the underlying supervised training heavily relies on binary labels and lacks access to fine-grained Chinese datasets with deep semantic annotations. This issue is particularly evident in dynamic social contexts such as Bilibili, which features a unique danmaku (bullet-screen comment) culture, existing datasets lack detailed topic classification and interpretation of true meanings. This results in insufficient prior knowledge of the underlying malicious motives of euphemisms during model learning, limiting the effectiveness of semantic representation learning.

### 2.2. Contrastive Learning for Implicit Semantics

Although dynamic word representations have significantly improved semantic modeling capabilities, relying solely on supervised learning and traditional sampling strategies often makes it difficult to construct high-quality annotated datasets covering diverse forms of euphemistic expressions [27,28,29]. Therefore, some studies have begun to introduce contrastive, self-supervised, and semi-supervised mechanisms to alleviate the issues of data scarcity and high annotation costs.

Regarding the evolution of general contrastive learning frameworks, in the field of computer vision, MoCo [30] and SimCLR [31] form a representative dual-track paradigm for contrastive learning, where MoCo introduced a momentum contrast mechanism to ensure the feature stability of the negative sample dictionary. The natural language processing field subsequently developed variants based on text characteristics. SimCSE [32] introduces latent space perturbations via dropout to construct positive sample pairs, improving the Spearman correlation coefficient by 23.6% over traditional methods in the STS-B semantic similarity task. Targeting supervised learning scenarios, Gunel et al. [33] proposed a joint loss function that combines cross-entropy and contrastive loss using a weighting coefficient, improving average accuracy by 2.9% on the GLUE benchmark. The more innovative DualCPC framework [34] achieved multi-granularity contrast through a two-level optimization strategy involving instance-level bidirectional prediction and cluster-level inter-cluster contrast, further improving model accuracy.

When applying the aforementioned theories to implicit euphemistic semantic detection, contrastive learning shows strong generalization potential. Kim et al. [12] proposed the contrastive learning method ImpCon, which shortens the representation distance between the “implicit meaning” and the corresponding post, encouraging the model to learn their semantic relationship, thereby enhancing cross-dataset generalization. Lu et al. [13] proposed a dual contrastive learning framework combining self-supervised contrastive learning with supervisory signals to capture high-level semantic information while addressing challenges such as data imbalance, subjectivity, and contextual dependency. Building upon this, Deng et al. [14] constructed the large-scale unsupervised dataset AugCOLD and employed a multi-teacher knowledge distillation method, where multiple independently trained teacher models provide soft labels for the student model, extracting supervisory signals from unlabeled data, thereby improving the model’s robustness and generalization. Meanwhile, some studies focus on the interpretability of the model’s decision-making process and the impact of training data on prediction results. For example, testing with Concept Activation Vectors has been used to evaluate the model’s sensitivity to specific semantic concepts, combined with data augmentation to enhance robustness against novel euphemisms [35]; TrackIn [15], a gradient-based method, was designed to analyze the contribution of training samples to the prediction results of test samples, improving model robustness from a data-centric perspective.

Despite the potential of contrastive learning in alleviating data scarcity, the existing mechanisms are highly susceptible to semantic collapse when processing harmful euphemisms. Because euphemisms exhibit highly similar surface forms but opposite polarities in normal versus malicious contexts, traditional single-view contrastive learning struggles to accurately capture such subtle semantic shifts, leading to semantic collapse and representation homogenization issues.

### 2.3. Enhancement Methods Fusing External Knowledge

Since the true meaning of text often depends on external background knowledge, domain conventions, and syntactic complexity, various research directions have incorporated external knowledge and explicit structural information to enhance detection performance.

In terms of explicit structure modeling and multimodal fusion, Pavlopoulos et al. [16] constructed the TOXICSPANS dataset, annotating harmful spans within text, and trained sequence labeling models based on this to achieve finer-grained, span-level identification of harmful expressions. Wang et al. [17] introduced adapter modules on top of pre-trained models to fuse multi-source features such as phonology, glyphs, and vocabulary into BERT. Huang et al. [18] proposed a two-stage framework that first generates euphemism candidates based on pre-trained language models, and then models the structural relationships between words using a syntactic dependency graph via syGCN, establishing mappings between euphemisms and their semantic interpretations, effectively improving both identification and interpretation capabilities.

On the other hand, to more fully utilize contextual settings and user-level information, Ghosh et al. [19] proposed CoSyn, a context-synergized neural network that jointly models dialogue context, user history, and social relationships to detect harmful euphemisms in online conversations, significantly improving detection accuracy in complex interactive scenarios. As internet content expands from pure text to modalities like memes, Zhang et al. [20] proposed a topology-aware optimal transport framework to model harmful euphemistic content in Internet memes, highlighting the importance of multimodal information for interpreting implicit expressions.

In expanding implicit reasoning capabilities, Prompt-based Chain-of-Thought (Prompt CoT) provides a promising direction for mining deep semantics. Jason Wei et al. [21] address challenges in complex reasoning tasks by introducing chain-of-thought prompting, confirming that this method is particularly effective on large-scale language models. Zhang et al. [36] explored the application of Automated Chain-of-Thought (Auto CoT) in large language models, aiming to improve reasoning ability by automatically generating reasoning steps; Kojima et al. [22] demonstrated the feasibility of zero-shot reasoning in LLMs to new tasks. However, external knowledge injection alone is insufficient to ensure reasoning accuracy. Zhou et al. [37] pointed out that irrelevant reasoning rationales can affect the model’s robustness; Wu et al. [38] further revealed through structural causal models that biased information in pre-training data might impair the reasoning capabilities of LLMs. To address these challenges, Zhao et al. [23] proposed KG-CoT, an innovative knowledge enhancement paradigm utilizing small-scale step-by-step graph reasoning models to reason over knowledge graphs, generating high-confidence reasoning chains for large-scale LLMs; Mondal et al. [24] proposed the KAM-CoT framework, which deeply integrates CoT reasoning, knowledge graphs, and multimodal features, providing comprehensive support for understanding multimodal harmful content while controlling computational costs. Chen et al. [39] proposed the SCCN framework for fake news detection, which effectively leverages social contextual cues.

Although existing methods attempt to utilize large models for knowledge injection, most remain at the stage of shallow text concatenation. Current research has yet to achieve organic fusion; it is unable to utilize the external knowledge of large models for high-confidence logical reasoning, nor can it adaptively avoid data homogenization in the feature space, which limits the upper bound of representation quality for harmful euphemism detection.

### 2.4. Multi-Task Learning and Debiasing Research

Multi-Task Learning (MTL), as an effective learning paradigm, improves model generalization by sharing knowledge across related tasks [40], and has been successfully applied in scenarios such as hate speech classification [41], dialogue system development [42], and sentiment analysis [43].

The core of multi-task learning lies in the design of the parameter sharing mechanism, which currently follows two main paths: hard sharing and soft sharing. Regarding hard sharing, models typically employ a unified bottom network supplemented by task-specific head modules, such as the BERT-based multi-task neural network proposed by Liu et al. [44]. Inspired by ensemble learning, Ma et al. [45] designed a Multi-gate Mixture-of-Experts (MMoE) model, where the shared bottom consists of multiple neural network experts, using gating mechanisms to control knowledge sharing, thereby effectively mitigating conflicts between tasks. Comparatively, soft sharing offers higher flexibility by sharing only a small number of parameters across tasks. For instance, Duong et al. [46] introduced regularization terms between task-specific networks to constrain parameter differences, enabling cross-task knowledge transfer while avoiding negative transfer from over-sharing.

At the task design level, multi-task learning has been introduced to strengthen the model’s modeling of multi-dimensional attributes. Flor et al. [47] found that when harmful category discrimination is jointly trained with related sub-tasks, the model can better distinguish different types of offensive euphemisms, improving overall detection performance across categories. Meanwhile, to alleviate bias issues in harmful content detection, Manzini et al. [48] approached the problem from the perspective of word embedding debiasing, proposing new bias evaluation metrics andconstructing a bias subspace via principal component analysis to robustly reduce bias risks in multi-classification tasks at the representation level. Such works provide a reference for introducing multi-task learning and fairness constraints for harmful euphemism detection.

However, despite current strategies like multi-task learning enriching the reference features of models, existing detection models still suffer from the fundamental limitation of single-dimensional perception in their architectural design. Most existing systems treat text as a single linear sequence for feature extraction, or merely rely on rigid feature concatenation and shallow bottom-layer parameter sharing during fusion. This coarse fusion strategy is prone to spurious correlations, which in turn leads to overfitting on high-frequency lexical patterns.

In summary, although detection models have evolved from static lexicons to dynamic representation learning, contrastive learning mechanisms, and multi-task frameworks, existing paradigms still encounter two fundamental bottlenecks when processing harmful euphemisms:

1. Single-Dimensional Perception and Shallow Feature Fusion: Existing traditional and lightweight frameworks predominantly rely on single-dimensional text sequences. Even when external features or multi-task sharing mechanisms are introduced, they are typically integrated through rigid and shallow concatenation. When confronted with highly obscure rhetorical disguises, this structural limitation inherently causes the aforementioned cognitive reasoning deficit, inevitably leading to semantic collapse. Consequently, traditional models frequently generate spurious correlations and overfit to high-frequency surface words, rather than structurally decouple underlying syntactic, contextual, and rhetorical anomalies.
2. Deployment Dilemmas and Shallow Integration of LLMs: Recently, LLMs have shown strong zero-shot reasoning capabilities via CoT prompting. However, current LLM-augmented methods typically inject external knowledge by appending generated reasoning rationales to prompts during online inference. This shallow, text-level fusion fails to organically reshape the model’s latent cognitive space. Furthermore, relying on massive LLMs for direct online inference is highly susceptible to pre-training biases and noisy rationales, while also incurring the prohibitive computational overhead and latency mentioned earlier. This makes them impractical for real-time moderation in dynamic, high-throughput social platforms.

## 3. Methodology

This section presents the dataset construction module, the representation learning module, and the detection model in detail.

Dataset Construction Module: This module is responsible for collecting comment data from Bilibili and annotating it according to predefined standards, thereby providing data support for subsequent research.

Representation Learning Module: The core function of this module is to effectively extract and model external knowledge from LLMs for harmful euphemisms, using deep prompt CoT and multi-head contrastive learning. This improves the quality of representation learning for harmful euphemisms and strengthens the semantic fidelity and expressiveness of the resulting representations.

Detection Model: This module first converts the input sentences into word vector representations to extract fundamental semantic information. Subsequently, a triple semantic perception module extracts syntactic, contextual, and rhetorical features, respectively. Finally, the extracted multi-dimensional semantic features are adaptively fused, and a classifier is used for final harmful euphemism detection.

### 3.1. Dataset Construction

During the data construction phase, we selected Bilibili, a major video-sharing platform in China, as the primary source of raw harmful euphemism data. The proposed dataset construction methodology encompasses the following stages:

**(1) Seed Keyword Selection.** To construct a comprehensive harmful euphemism dataset for social networks, we designed the data collection pipeline based on the principles of topic diversity and category balance. Initially, we manually identified four primary categories of seed keywords: political content, vulgar language, general insults, and targeted group attacks. The representative examples of the initial seed keywords are presented in Table 2.

Subsequently, based on these seed keywords, we used the Bilibili search engine to select seed videos from diverse content categories, ensuring the diversity of comment sources. A web crawler was deployed to collect comments in batches from the corresponding video sections. Simultaneously, video popularity was evaluated using metrics such as view counts, likes, coin donations, comments, and shares, to ensure the representativeness and broad coverage of the dataset.

**(2) Data Crawling and Bias Control.** We employed a Breadth-First Search (BFS) strategy for data collection. First, to control data bias and avoid bias toward a single “echo chamber”, we implemented stratified sampling across diverse Bilibili video partitions (e.g., news, entertainment, gaming, and lifestyle), extracting comments from target videos and storing them in a database.

Notably, to capture the temporal evolution of emerging harmful content on contemporary internet platforms, we did not rely on a static search strategy. Instead, we dynamically selected and updated seed videos based on popularity metrics such as views, likes, and trending indices. Furthermore, time-sensitive variants, such as meme-based expressions, were incorporated. During BFS traversal, the crawler calculated video popularity and selected the top 10 recommended videos as child nodes for the next iteration, while performing exact-match deduplication to remove redundant nodes. Over four traversal iterations, the crawler collected approximately 8 million comments from 5000 videos. Next, to ensure data quality and establish rigorous inclusion criteria, we filtered the raw comment corpus using the initial seed dictionary and applied a length filter to remove comments shorter than two characters. Ultimately, following rigorous manual annotation and multiple rounds of expert review, we constructed a high-quality Bilibili comment dataset consisting of approximately 26,517 samples.

**(3) Data Annotation.** To improve annotation accuracy and mitigate subjective bias, we carefully designed the annotation workflow. First, to ensure diversity in perspectives, we recruited annotators of different genders and regional cultural backgrounds, and provided each participant with systematic training on euphemistic contexts. Second, rather than imposing fixed annotation rules, our annotation guidelines were iteratively formulated through progressive analysis of the collected Bilibili corpus. We conducted several rounds of small-scale pilot annotations and intensive discussions of edge cases to refine fine-grained annotation standards.

During the formal annotation phase, three core principles were strictly enforced: (1) annotators must independently execute their tasks based on the established guidelines; (2) post-annotation consistency must be verified by an independent third party; and (3) highly inconsistent annotations require mandatory re-evaluation. Acknowledging that cultural backgrounds induce variations in comprehending harmful euphemisms, annotators were required to interpret euphemistic terms strictly based on their objective contextual scenarios. Furthermore, independent evaluation and tagging of euphemism labels were mandatory. Finally, random spot checks were conducted to enforce quality control and ensure annotation consistency. Major discrepancies in euphemism labels were resolved through collaborative team reviews and consensus discussions. To quantitatively evaluate and verify annotation reliability, we calculated the Fleiss’ Kappa statistic across the entire dataset. The overall coefficient reached 0.82, indicating substantial agreement among annotators and ensuring the high quality of the dataset.

Based on this rigorous dataset construction strategy, we observed that harmful euphemisms distort normal, healthy discourse through means such as morphological variations, metaphors, and sarcasm to convey harmful information like racial discrimination, child pornography, and group hatred. Based on the analysis of the dataset samples, their characteristics can be summarized as follows: (1) the intent of the comment is inconsistent with its literal interpretation; (2) the use of uncommon phrases or combinations of special characters and numbers; (3) a noticeable shift in tone or emotion compared to the normal context; (4) seemingly illogical yet contextually consistent expressions.

Detailed statistics of the constructed HECD dataset are presented in Table 3. It contains 26,517 samples, covering 4 topic types and 8 different attack methods of harmful euphemisms, providing fine-grained annotations for subsequent research on harmful euphemism detection and identification.

As shown in Table 3, the HECD dataset exhibits a moderate class imbalance between harmful (11,481) and harmless (15,036) samples, with harmless data comprising 56.7% of the corpus. This ratio of approximately 1:1.31 reflects the natural distribution of social media content, where benign expressions are generally more prevalent. This moderate imbalance remains within a controllable range and does not constitute a severe skew that would dominate classifier behavior. Furthermore, an uneven distribution is observed at the topical level, with categories such as “Group Attacks” containing more samples than “Political Content” and “Everyday Insults”. While such topical imbalance may bias training toward high-frequency topics and affect classification accuracy, we deliberately preserved this real-world distribution. Because the topical differences do not represent an order-of-magnitude skew, artificial re-balancing was avoided to prevent the introduction of synthetic noise.

### 3.2. Representation Learning

**Figure 1:**
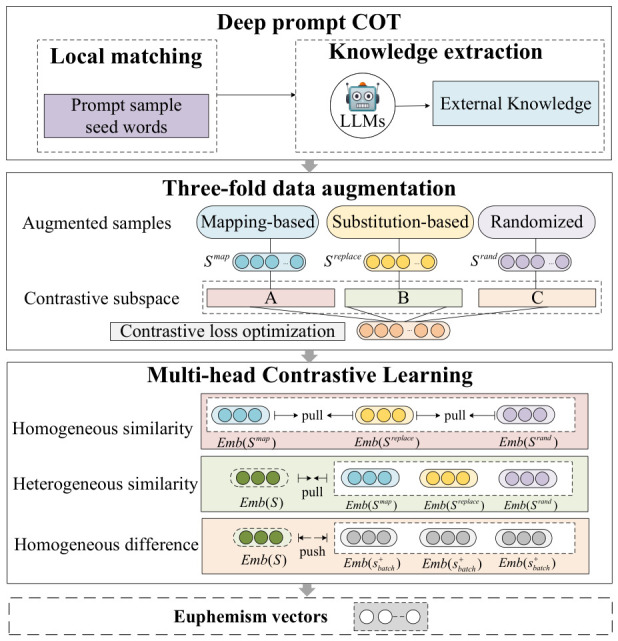
Schematic diagram of the harmful euphemism representation learning framework based on deep prompt chain-of-thought and multi-head contrastive learning.

To address the issues of inaccurate representation and insufficient generalization of harmful euphemisms, we propose a harmful euphemism Representation Learning Framework via Deep Prompt COT and Multi-head Contrastive Learning (HER-MCL). This framework is a representation learning method for harmful euphemistic semantics that integrates the generalized knowledge of large models with self-supervised learning, enabling a more accurate extraction of semantic representation vectors of harmful euphemisms from pre-trained corpora, as illustrated in Figure 1.

#### 3.2.1. Seed Lexicon Matching Stage

To enhance the ability of LLMs to mine external knowledge of harmful euphemisms, we design a seed word prompt sample selection algorithm based on an adaptive threshold, integrating the semantic relationship between harmful euphemisms and their corresponding explanatory words for harmful meanings.

Suppose the local harmful euphemism lexicon set is *O*, where $o_{i}$ represents a word in the local harmful euphemism lexicon. Additionally, there are corresponding explanatory words for their harmful meanings, denoted as $e_{i}$. For each seed word and its corresponding explanatory word, their vector representations are obtained. Cosine similarity is used to measure their similarity, while an adaptive threshold is employed to ensure that the seed words accurately represent their harmful meanings. Based on the calculated adaptive threshold, a set of seed words highly correlated with the target seed words and mapped words is filtered out. Finally, an iteratively optimized seed word set is output, containing seed words that are highly relevant to the target harmful euphemisms and possess diversity, capable of comprehensively covering the semantic space of the selected terms.

#### 3.2.2. Chain-of-Thought Template Generation Stage

The deep prompt CoT refers to a prompt template generated by LLMs, which integrates prior knowledge and encodes a complete and structured reasoning process.

First, an initial CoT template is constructed based on a locally curated harmful euphemism lexicon and its associated metadata. Second, the design of the template incorporates the proposed five cognitive dimensions of harmful euphemisms, namely: topic category, attack strategy, reasoning chain, attack variant, and structured mapping relations. These elements are introduced to ensure that the reasoning process aligns with the cognitive characteristics of euphemistic expressions. Third, the initial template is further refined and expanded through interaction with an open-source LLM API. During this stage, both the extracted local knowledge and the five cognitive dimensions are jointly integrated and enriched, resulting in a more comprehensive and structurally grounded deep CoT template. Finally, the generated deep CoT template guides the LLM in effectively eliciting auxiliary external knowledge from the original harmful euphemistic expressions, thereby enhancing semantic enrichment and implicit intent extraction.

In this section, we define the design principles of the deep CoT template and specify the objective of each reasoning stage. The corresponding template structures for different reasoning stages are presented in Table 4.

#### 3.2.3. Three-Fold Data Augmentation Stage

The goal of this stage is to generate multiple similar samples that are semantically close to the original sentence but differ in surface form, thereby enhancing the model’s robustness against harmful euphemism noise and its variations, and reducing the risk of overfitting during representation training. The specific methods are as follows:

(1) Mapping-based Augmentation: The original harmful euphemisms in the samples are replaced with their corresponding harmful meaning mapped words from the lexicon, generating similar augmented samples of the original harmful euphemisms.
(2) Substitution-based Augmentation: Utilizing the morphological variants of harmful euphemisms generated by the deep prompt CoT, the original harmful euphemisms in the sentence samples are replaced with these variants to generate similar augmented samples.
(3) Randomized Augmentation: Three randomization strategies commonly used in traditional contrastive learning frameworks are comprehensively applied, namely dropout, cutoff (dimensional clipping), and character shuffling.

#### 3.2.4. Multi-Head Contrastive Learning Stage

Multi-head Contrastive Learning (MCL) utilizes a pre-trained language model and multi-dimensional projection heads to encode harmful euphemisms into token embedding representations. These representations are then projected by multiple heads into low-dimensional vector spaces at different hierarchical levels. Ultimately, contrastive loss is applied across projection subspaces to bring similar samples closer and push dissimilar ones apart. Specifically, MCL constructs positive and negative pairs through a three-part data augmentation scheme. The positive samples consist not only of the original sentences but also substitution-based augmented samples generated via the Deep Prompt Chain of Thought and the generalized knowledge of large language models, mapping-based augmented samples derived from explicit harmful meanings, and randomized augmented samples generated by three randomization strategies. This enables the model to learn consistent semantics across different views. Conversely, negative samples are drawn from other instances within the same mini-batch, which reinforces the model’s discriminative capacity between distinct semantics.

By minimizing distances between positive pairs and maximizing distances to negative samples, MCL achieves self-supervised optimization of semantic representations. A dynamic weight adjustment mechanism is also introduced, enabling semantic differences across various subspaces to be adaptively balanced during training. This ensures that each projection head is fully utilized, yielding representation vectors that are both semantically rich and accurate. The framework is trained end-to-end, jointly optimizing the three types of contrastive samples via backpropagation, thereby improving the model’s accuracy and robustness in capturing the implicit information concealed within harmful euphemisms.

The MCL stage employs a multi-head projection architecture. To maintain structural symmetry with the 12 inherent self-attention heads of the foundational pre-trained textual encoder (RoBERTa), this framework precisely configures 12 projection heads. These 12 projection heads are systematically partitioned into 3 groups of projection subspaces with varying dimensions (4 heads per group), mapping the sentence representation matrices obtained from the pre-trained encoder into multidimensional vector subspaces for contrastive optimization. Let the sentence representation output by the pre-trained encoder be denoted as $x\in R^{L\times d_{\mathrm{encoder}}}$, where $d_{\mathrm{encoder}}$ is the hidden layer dimension. For each projection head h∈{1,2,…,12}, a linear projection is applied:

(1) $$p^{h}=W^{h}\bar{x}$$

where $\bar{x}=\frac{1}{L}\sum_{i=1}^{L}x_{i}$ represents the sentence-level mean-pooled representation, and the specific projection dimension for a given group is denoted as $d_{h}$. To ensure training stability and prevent gradient vanishing or exploding during the initial stages of contrastive optimization, the weight matrices $W^{h}$ of all 12 projection heads are strictly initialized using Xavier Uniform initialization.

These 12 projection heads are not pre-assigned to specific data augmentation types (i.e., mapping-based, substitution-based, or randomized augmentations). Instead, all 12 heads globally process the representations of both the original sentence and its three corresponding augmented views simultaneously. This non-assignment strategy is designed: pre-assigning heads would artificially isolate linguistic features and restrict the receptive field of each subspace. By allowing every head to globally compute distances across all three augmentation types, the framework relies entirely on the contrastive loss and the dynamic weight adjustment mechanism to implicitly and organically decouple the multi-dimensional rhetorical disguises across the 12 orthogonal latent subspaces.

Within each projection space, the relationships among samples are evaluated through three distance metrics: heterogeneous similarity distance, homogeneous similarity distance, and homogeneous difference distance. These correspond to the average Euclidean distance between the original sentence and its three augmented samples, the average distance among the three augmented samples themselves, and the average distance between the original sentence and the set of negative samples.

Heterogeneous similarity distance metric:

(2) $$d_{\mathrm{het}}=\frac{1}{3}\sum_{i=1}^{3}{∥p_{\mathrm{orig}}-p_{\mathrm{sim},i}∥}_{2}$$

Homogeneous similarity distance metric:

(3) $$d_{\mathrm{hom}}=\frac{1}{3}\sum_{i<j}{∥p_{\mathrm{sim},i}-p_{\mathrm{sim},j}∥}_{2}$$

Homogeneous difference distance metric:

(4) $$d_{\mathrm{diff}}=\frac{1}{N}\sum_{n=1}^{N}{∥p_{\mathrm{orig}}-p_{\mathrm{neg}}^{\left(n\right)}∥}_{2}$$

Since the similarity between the heterogeneous and homogeneous similarity distances should be greater than that between the heterogeneous and homogeneous difference distances, these three distance metrics satisfy the constraint $|d_{\mathrm{hom}}-d_{\mathrm{het}}\left|<\right|d_{\mathrm{het}}-d_{\mathrm{diff}}|$. Therefore, we introduce a dynamic weight function for distance metrics to ensure that the similarity margin among the three different distance metrics is maintained within a reasonable range. This dynamic weight is calculated using the Sigmoid function:

(5) $$w_{\mathrm{sim}}=\sigma \left(\alpha \left(d_{\mathrm{hom}}-d_{\mathrm{het}}\right)\right)$$

(6) $$w_{\mathrm{diff}}=\sigma \left(\alpha \left(d_{\mathrm{het}}-d_{\mathrm{diff}}\right)\right)$$

where α is a scaling coefficient and σ(·) represents the Sigmoid function. The contrastive loss for a single projection head is defined as:

$$\Gamma^{\left(h\right)}=E\left[w_{\mathrm{sim}}\cdot d_{\mathrm{hom}}+w_{\mathrm{diff}}\cdot d_{\mathrm{diff}}\right]$$

To achieve multi-dimensional rhetorical features, the final global loss is formulated as the average of the losses from all 12 projection heads aligned with the RoBERTa attention mechanism:

(7) $$\Gamma^{\mathrm{total}}=\frac{1}{12}\sum_{h=1}^{12}\Gamma^{\left(h\right)}$$

Using 12 projection heads aligned with the backbone enables contrastive learning to capture representations across multiple dimensions. This architecture allows for the independent fine-tuning of specific attention patterns and the capture of finer-grained semantic nuances. Furthermore, the dynamic weight mechanism guarantees an equilibrium between homogeneous similarity and heterogeneous difference, successfully pulling similar samples closer while effectively discriminating negative samples, thereby constructing a more robust and discriminative representation space.

#### 3.2.5. Representation Learning Training Workflow

This subsection describes the overall representation learning process from data construction to encoder-based representation generation. The overall training procedure of the harmful euphemism representation learning framework, which is based on a deep prompt CoT and multi-head contrastive learning, is summarized as follows:

(1) Input Preprocessing and Knowledge Matching: We first construct training samples of harmful euphemisms. These samples are matched against a local harmful euphemism dictionary to extract local sensitive vocabulary and related knowledge. This process constructs a structured corpus containing the original sentence, euphemism, and its semantic interpretation.
(2) Deep Prompt Chain-of-Thought Generation: Based on a cognition-driven prompt design, we construct a CoT template. An LLM is then used to complete the CoT template and leverage external knowledge for the harmful euphemisms based on this final template. Structured auxiliary information is extracted according to this chain, representing the original sentence, euphemism, semantic interpretation, transformed variants, CoT reasoning, and attack type. This stage provides guidance for external knowledge augmentation for subsequent representation learning.
(3) Contrastive Learning Sample Construction: A pre-trained language model encodes the original sentences to obtain base semantic representations. Subsequently, the acquired auxiliary knowledge of harmful euphemisms is used to construct contrastive pairs.
(4) Multi-Head Contrastive Learning Training: The enhanced inputs are passed through 12 projection heads, mapping them into sub-spaces of different dimensions. After constructing positive and negative pairs, the multi-head contrastive learning module calculates the contrastive loss within each sub-space. A dynamic weighting mechanism is applied to adjust the various distances, yielding a comprehensive contrastive loss. Next, the Adam optimizer is utilized to optimize this final loss, thereby updating the parameters across all layers of the model. This enables the semantic encoder to achieve optimized representations for euphemism detection.
(5) Enhanced Semantic Vector Output: After freezing the encoder parameters, the text of harmful euphemisms is fed into the model, and the encoder outputs enhanced semantic vectors through forward propagation. The harmful euphemism feature vectors, optimized through this representation learning process, can be directly used for downstream tasks, such as harmful euphemism detection and harmful word recognition.

### 3.3. Detection Model

**Figure 2:**
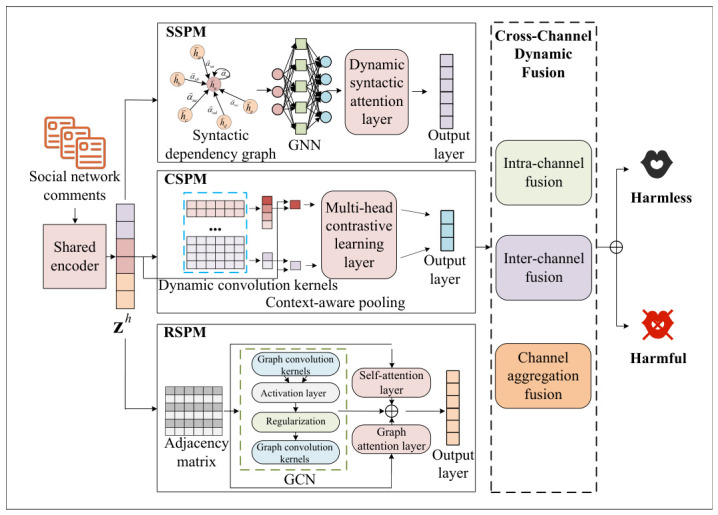
Harmful Euphemism Detection Framework Based on Multi-dimensional Semantic Perception Fusion.

We propose HED-MSP, a harmful euphemism detection framework based on multi-dimensional semantic perception and cross-channel adaptive fusion. Its design is motivated by the linguistic differences between harmful euphemisms and explicit harmful expressions. Unlike explicit content, euphemisms rely on multi-layered semantic obfuscation, posing three key challenges: formal variations (e.g., homophones and abbreviations) that disrupt syntax, contextual ambiguity where malicious intent is hidden beneath neutral semantics, and rhetorical encoding (e.g., metaphors and irony) conveying non-literal malicious intent. To address these challenges, HED-MSP consists of three modules—the Syntactic Semantic Perception Module (SSPM) for dependency modeling, the Contextual Semantic Perception Module (CSPM) for context-aware disambiguation, and the Rhetorical Semantic Perception Module (RSPM) for rhetorical semantic modeling—along with a cross-channel adaptive fusion module. This multi-dimensional design decouples semantic heterogeneity across features, preventing interference and robustness degradation in shallow feature fusion, as illustrated in Figure 2.

#### 3.3.1. Semantic Perception Modules

(1) Syntactic Semantic Perception Module

The syntactic–lexical semantics of harmful euphemisms capture relationships between euphemistic expressions and normal lexical items, as well as their syntactic structures. In benign texts, syntactic relations typically follow standard grammatical rules. However, harmful euphemisms often deviate from normal word order and grammatical structure. Therefore, a deeper analysis and modeling of syntactic dependency patterns and sentence component features in harmful euphemisms can significantly improve both the accuracy and generalization ability of detection models.

Specifically, SSPM leverages dependency parsing, graph neural networks, and graph attention mechanisms to model syntactic dependencies encoding euphemistic and harmful signals. SSPM consists of an input graph construction layer, a graph neural network layer, a syntactic attention layer, and an output layer.

**a. Input Graph Construction Layer.** Given an input sentence $S=w_{1},w_{2},\ldots ,w_{n}$, a pre-trained model (e.g., BERT) generates word embeddings $E=e_{1},e_{2},\ldots ,e_{n}$. A dependency parser (e.g., spaCy) is used to construct a dependency graph:

(8) G={V,E}

Here, *V* represents all word nodes within the sentence, including the harmful euphemistic terms. *E* denotes the set of dependency edges, which serve to characterize the syntactic relationships between words. A dependency parsing tool such as spaCy (zh_core_web_sm) is employed to construct the syntactic dependency graph. We adopt spaCy for dependency parsing due to its efficiency and robustness on social media text. Compared with parsers trained exclusively on formal newswire corpora, spaCy provides more robust baseline performance on heterogeneous and informal textual inputs commonly observed in social media scenarios. In addition, its lightweight design enables efficient large-scale processing, which is critical for real-world toxic content analysis tasks.

Node features are initialized using word embeddings $x_{i}$, while edge features $e_{ij}$ are represented through dependency relation embeddings.

**b. Graph Neural Network Layer.** This layer encodes dependency relations of euphemistic expressions using a Graph Attention Network (GAT), where each node aggregates information from its neighbors via learned attention weights:

(9) $$h_{i}^{l+1}=\sigma \left(\sum_{j\in N(i)}\alpha_{ij}W^{\left(l\right)}h_{j}^{\left(l\right)}\right)$$

The attention weight $\alpha_{ij}$ is computed via an attention mechanism. $W^{\left(l\right)}$ denotes a learnable parameter matrix. N(i) denotes the neighborhood of node *i*.

Subsequently, multi-layer graph neural networks extract both local and global syntactic features, followed by feature concatenation:

(10) $$h_{i}^{\mathrm{multi}}=\mathrm{Concat}\left(h_{i}^{1},h_{i}^{2},\ldots ,h_{i}^{L}\right)$$

**c. Dynamic Syntactic Attention Layer.** To better capture key syntactic features, a dynamic syntactic attention mechanism is introduced. This mechanism assigns importance weights to euphemistic nodes based on their roles within the syntactic graph.

(11) $$\beta_{i}=\mathrm{softmax}\left(W_{\mathrm{syntax}}h_{i}^{\mathrm{multi}}\right)$$

The attention layer dynamically adjusts the contribution of each node, and the syntactic–lexical semantic feature vector is updated by combining node features with attention weights.

(12) $$h_{\mathrm{syntax}}=\sum_{i}\beta_{i}h_{i}^{\mathrm{multi}}$$

**d. Output Layer.** The syntactic features $h_{\mathrm{syntax}}$ are concatenated with contextual embeddings $h_{\mathrm{context}}$ to form the final output vector $h_{\mathrm{enhanced}}$:

(13) $$h_{\mathrm{enhanced}\_\mathrm{syntax}}=\mathrm{Concat}\left(h_{\mathrm{syntax}},h_{\mathrm{context}}\right)$$

This vector encodes syntactic–lexical information of harmful euphemisms, enabling the model to capture discriminative syntactic patterns and improve classification performance.

(2) Contextual Semantic Perception Module

Contextual semantics refer to surrounding information of harmful euphemisms, including topic and discourse-level cues. On social platforms, harmful meanings are often recognized only when euphemistic expressions deviate from their contextual semantics, leading to a cognitive shift.

To model this phenomenon, CSPM adopts a context-aware dynamic pooling mechanism. By capturing semantic features and contextual variations, this module improves detection accuracy. It consists of an input encoding layer, a dynamic convolution learning layer, a context-aware pooling layer, and an output layer.

**a. Input Encoding Layer.** Each word in the input text $T=w_{1},w_{2},\ldots ,w_{n}$ is encoded into a vector representation using a pre-trained encoder:

(14) $$E=\{e_{1},e_{2},\ldots ,e_{n}\}$$

Here, $e_{i}$ denotes the embedding of the word $w_{i}$.

**b. Dynamic Convolution Learning Layer.** Dynamic convolution kernels are generated to capture contextual semantics at different granularities. In CSPM, this layer is implemented as a lightweight multi-scale convolution mechanism.

We use three parallel convolution pathways with kernel sizes w∈1,3,5. This multi-scale design allows the model to capture features across different linguistic granularities, including unigram-level lexical cues (w=1), phrase-level syntactic structures (w=3), and short-context semantic patterns (w=5).

To ensure efficient inference, each convolution kernel is generated via a Multi-Layer Perceptron (MLP) conditioned on $E_{i}$:

(15) $$K_{i}^{\mathrm{dynamic}}=\mathrm{MLP}\left(E_{i}\right)$$

Here, $K_{i}^{\mathrm{dynamic}}$ denotes generated convolution kernels. $E_{i}$ denotes feature representations. For each kernel size, we adopt a compact configuration of 8 convolution filters, resulting in a total of 24 dynamic convolution filters across the three parallel branches. The convolution operations extract contextual representations:

(16) $$H_{\mathrm{conv}}=\sum_{i}K_{i}^{\mathrm{dynamic}}\cdot E_{i}$$

Here, $K_{i}^{\mathrm{dynamic}}$ denotes the extracted features. $E_{i}$ is the original input. $H_{conv}$ is the resulting feature vector. This configuration captures semantic shifts in implicit euphemistic expressions while avoiding parameter explosion in standard dynamic convolution, balancing representational capacity and efficiency.

**c. Context-Aware Pooling Layer.**

This layer introduces an attention-based pooling mechanism that assigns weights to each word:

(17) $$\alpha_{i}^{\mathrm{dynamic}}=\mathrm{softmax}\left(W_{\mathrm{att}}E_{i}+b_{\mathrm{att}}\right)$$

Here, $\alpha_{i}^{\mathrm{dynamic}}$ denotes the attention weight of the i-th word. $W_{\mathrm{att}}$ and $b_{\mathrm{att}}$ are learnable parameters. Weighted pooling is then applied to obtain a global representation:

(18) $$H_{\mathrm{pooled}}=\sum_{i}\alpha_{i}^{\mathrm{dynamic}}\cdot E_{i}$$

Here, $E_{i}$ denotes the initial input of harmful euphemistic features.

**d. Output Layer.**

The pooled feature is fed into the output layer as the final representation:

(19) $$h_{\mathrm{enhanced}\_\mathrm{syntax}}=\mathrm{softmax}\left(W_{\mathrm{out}}H_{\mathrm{pooled}}+b\right)$$

This vector $h_{\mathrm{enhanced}\_\mathrm{syntax}}$ encodes both local and global contextual information, improving the model’s ability to detect harmful euphemisms.

(3) Rhetorical Semantic Perception Module

Harmful euphemisms often employ rhetorical strategies to disguise their original intent and evade detection systems. This phenomenon, referred to as rhetorical behavior, necessitates enhanced semantic perception capabilities for accurate classification.

The Rhetorical Semantic Perception Module (RSPM) aims to identify and model the impact of rhetorical strategies on textual semantics. This includes implicit semantic transformation and rhetorical device analysis. The module integrates a Rhetoric Identification Network (RIN), a semantic reasoning layer, and multi-level self-attention mechanisms, along with graph neural networks for local structural learning and contrastive learning for improved discriminative power.

The module consists of an input layer, a graph convolutional network layer, a multi-level self-attention layer, a semantic reasoning layer, and a rhetorical feature fusion layer.

**a. Input Layer.** The input text $X=\{x_{1},x_{2},\ldots ,x_{n}\}$ is encoded into embeddings $E=\{e_{1},e_{2},\ldots ,e_{n}\}$. These embeddings are passed through a GCN layer to capture rhetorical structures, a self-attention layer to model word importance, a semantic reasoning layer to extract inferential relations, and a fusion layer to combine features.

**b. Graph Convolutional Network Layer.** This layer captures local rhetorical structures by modeling syntactic and logical relationships among words. Each word is treated as a node, and their relationships are represented by an adjacency matrix.

The node representation is updated as:

(20) $$P^{\left(k\right)}=\sigma \left(\hat{A}P^{\left(k-1\right)}W^{\left(k\right)}\right)$$

Here, $P^{k}$ denotes node features at layer *k*. $\hat{A}=D^{-\frac{1}{2}}AD^{-\frac{1}{2}}$ is the normalized adjacency matrix. $W^{\left(k\right)}$ is the learnable weight matrix. σ is the activation function. Through multiple layers, the model captures rhetorical patterns such as irony, metaphor, and exaggeration.

**c. Self-Attention Mechanism Layer.** This layer models the importance of each word in capturing rhetorical semantics. Query, key, and value representations are computed, and attention weights are applied:

(21) $$\mathrm{Attention}\left(Q,K,V\right)=\mathrm{softmax}\left(\frac{QK^{T}}{\sqrt{d_{k}}}\right)V$$

Here, $W_{Q}$, $W_{K}$, and $W_{V}$ are projection matrices, and $d_{k}$ denotes the dimensionality. This enables the model to capture global rhetorical dependencies.

**d. Semantic Reasoning Layer.** A Graph Attention Network (GAT) is used to model inferential relationships:

(22) $$\alpha_{ij}=\frac{\exp(\mathrm{LeakyReLU}\left(a^{T}\left[Wh_{i}\;\|\;Wh_{j}\right]\right))}{\sum_{k\in N(i)}\exp\left(\mathrm{LeakyReLU}\left(a^{T}\left[Wh_{i}\;\|\;Wh_{k}\right]\right)\right)}$$

Attention weights are computed between nodes and their neighbors. Node representations are updated via weighted aggregation:

(23) $$h_{i}^{\prime }=\sum_{j\in N(i)}\alpha_{ij}Wh_{j}$$

This allows the model to capture reasoning patterns embedded in rhetorical expressions.

**e. Rhetorical Feature Fusion Layer.** Features from the GCN layer, self-attention layer, and semantic reasoning layer are fused. Let the feature representations be $h_{\mathrm{GCN}}$, $h_{\mathrm{Att}}$ and $h_{\mathrm{GAT}}$, respectively. The final representation is computed as a weighted combination:

(24) $$h_{\mathrm{enhanced}\_\mathrm{rhetoric}}=\alpha_{1}h_{\mathrm{GCN}}+\alpha_{2}h_{\mathrm{Att}}+\alpha_{3}h_{\mathrm{GAT}}$$

Here, $\alpha_{1}$, $\alpha_{2}$, and $\alpha_{3}$ are all learnable coefficients. Following model training and convergence, these three learnable coefficients ultimately stabilize at: $\alpha_{1}=0.35$ (GCN features), $\alpha_{2}=0.20$ (self-attention features), and $\alpha_{3}=0.45$ (GAT features). This weight distribution possesses a high degree of interpretability. First, the GAT features carry the highest weight, indicating that dynamic structural relationships between nodes are the most discriminative factor when identifying rhetorical disguises such as metaphors and irony. Since rhetorical devices often rely on anomalous syntactic collocations within specific contexts, GAT’s adaptive edge-weighting mechanism is able to precisely capture these localized structural shifts. Second, the GCN features provide the model with globally stable topological structural anchors. Finally, the self-attention features carry the lowest weight. This further suggests that the disguise employed by harmful euphemisms relies heavily on structure rather than on purely lexical semantics. Without the structural constraints provided by graph neural networks, a purely semantic attention mechanism would be highly susceptible to being misled by the superficially innocuous vocabulary of euphemisms. Overall, this fused representation encodes both local and global rhetorical–semantic information, thereby enhancing the model’s capability to classify harmful euphemisms.

#### 3.3.2. Cross-Channel Dynamic Adaptive Fusion Method

We propose a Cross-channel Dynamic Adaptive Fusion (CDAF) method for efficient feature fusion via dynamic channel attention. It extracts multi-level semantic information of harmful euphemisms, improving detection accuracy and generalization.

(1) Input Layer

The input layer consists of three feature channels: lexical ($U_{\mathrm{syntax}}$), contextual ($U_{\mathrm{context}}$), and rhetorical ($U_{\mathrm{rhetoric}}$) features. These channels encode semantic information from different perspectives.

(2) Adaptive Multi-channel Attention Mechanism Layer

The adaptive multi-channel attention mechanism includes three components: intra-channel, inter-channel, and aggregation attention. These are used respectively to adjust the weights of features within each channel, adjust relationships between different channels, and adaptively fuse information from the three channels. Taking the lexical semantic feature channel as an example:

Intra-channel attention assigns weights to features within each channel:

(25) $${\mathrm{ATT}}_{\mathrm{intra}\_\mathrm{syntax}}=\mathrm{softmax}\left(W_{\mathrm{intra}}\cdot e_{\mathrm{syntax}}\right)$$

where $W_{\mathrm{intra}}$ is the weight matrix used for intra-channel features, generating the attention weight for each feature vector through an activation function.

Inter-channel attention models the relationship between different channels. By calculating the similarity between channels, it allocates weights to each channel:

(26) $${\mathrm{ATT}}_{\mathrm{context}\_\mathrm{syntax}}=\mathrm{sigmoid}\left(W_{\mathrm{inter}}\cdot \left[e_{\mathrm{context}},e_{\mathrm{syntax}}\right]\right)$$

where $W_{\mathrm{inter}}$ is the weight matrix used for inter-channel features. The attention value determines the relationship between channels, and the model can adaptively adjust the influence of each channel during fusion based on this information. Channel aggregation attention considers the joint relationship between intra-channel and inter-channel attention, generating the final attention weights for each channel and determining the contribution of each channel to the final fusion result:

(27) $${\mathrm{ATT}}_{\mathrm{syntax}\_\mathrm{sum}}=\sigma \left({\mathrm{ATT}}_{\mathrm{syntax}}+{\mathrm{ATT}}_{\mathrm{syntax}\_\mathrm{context}}+{\mathrm{ATT}}_{\mathrm{syntax}\_\mathrm{rhetoric}}\right)$$

where σ is the activation function, generating the final weight value based on the adaptive importance of each channel.

(3) Dynamic Parameter Fusion Layer

The dynamic parameter fusion layer performs weighted fusion of the features based on the three attention weights ${\mathrm{ATT}}_{\mathrm{syntax}\_\mathrm{sum}},{\mathrm{ATT}}_{\mathrm{context}\_\mathrm{sum}},{\mathrm{ATT}}_{\mathrm{rhetoric}\_\mathrm{sum}}$ generated by the adaptive multi-channel attention mechanism. The goal of this layer is to adjust the contributions of different channels during the fusion process using the weight information produced by the attention mechanism:

(28) $$h_{\mathrm{fusion}}=\left[{\mathrm{ATT}}_{\mathrm{syntax}\_\mathrm{sum}}\cdot e_{\mathrm{syntax}},{\mathrm{ATT}}_{\mathrm{context}\_\mathrm{sum}}\cdot h_{\mathrm{context}},{\mathrm{ATT}}_{\mathrm{rhetoric}\_\mathrm{sum}}\cdot h_{\mathrm{rhetoric}}\right]$$

This fusion process adjusts the contribution of different channels based on the attention weight of each channel, generating the final fused feature vector $h_{\mathrm{fusion}}$.

(4) Output Layer

Finally, the output layer calculates the harmful euphemism loss based on the feature vectors generated by the fusion of the above network structures:

(29) $$Loss_{\mathrm{toxic}}=-\frac{1}{N}\sum_{i=1}^{N}\left[y_{i}^{\mathrm{euph}}\log p_{i}^{\mathrm{euph}}+\left(1-y_{i}^{\mathrm{euph}}\right)\log\left(1-p_{i}^{\mathrm{euph}}\right)\right]$$

(30) $$Loss_{\mathrm{topic}}=-\frac{1}{N}\sum_{i=1}^{N}\sum_{t=1}^{T}y_{i,t}^{\mathrm{topic}}\log P_{i,t}^{\mathrm{topic}}$$

(31) $$Loss_{\mathrm{rhet}}=-\frac{1}{N}\sum_{i=1}^{N}\sum_{r=1}^{R}y_{i,r}^{\mathrm{rhet}}\log P_{i,r}^{\mathrm{rhet}}$$

where $Loss_{\mathrm{toxic}}$, $Loss_{\mathrm{topic}}$, and $Loss_{\mathrm{rhet}}$ are the losses for the harmful euphemism label, the attack topic label, and the rhetorical behavior label, respectively. Among them, the attack topic label and the rhetorical behavior label are annotated through the harmful euphemism seed lexicon and participate in model training as auxiliary features for the harmful euphemism detection task. Therefore, during model testing, only the results of the harmful euphemism label are calculated to compile metrics such as accuracy and recall for the harmful euphemism detection model.

## 4. Experiments

### 4.1. Experimental Setup

#### 4.1.1. Dataset

In this study, the HECD dataset is adopted as the benchmark for the harmful euphemism detection task and serves as the evaluation basis for both the representation learning framework HER-MCL and the detection framework HED-MSP. Specifically, HECD is split into training, validation, and test sets with a 7:1:2 ratio, ensuring a standardized and fair evaluation protocol for model training and assessment.

In addition, we construct a semantic textual similarity dataset, HEC-STS (Harmful Euphemism Comment Semantic Textual Similarity), to evaluate the quality of sentence embeddings from a semantic similarity perspective. This dataset is designed to assess how well sentence representations capture semantic information in the embedding space, rather than being used for downstream detection tasks.

HEC-STS is constructed from harmful euphemisms and their corresponding explanatory terms, which capture their underlying toxic meanings. Sentence pairs are generated by combining these euphemisms with their semantic interpretations. Each sample consists of a sentence pair of similar length. The pair is annotated with a semantic similarity score ranging from 0 to 5, which reflects the degree of semantic relatedness between the two sentences.

During evaluation, cosine similarity between sentence embeddings is computed and compared with human-annotated similarity scores. If the model-generated similarity distribution aligns closely with the annotated distribution, it indicates that the model better captures sentence-level semantics and therefore has stronger representation capability. Conversely, larger discrepancies suggest limitations in semantic modeling, leading to weaker representational quality of the learned embeddings.

#### 4.1.2. Baselines

To evaluate the effectiveness of the proposed HED-MSP model, we compare its performance against a diverse set of representative baselines, spanning traditional text classification models, pre-trained language models, and state-of-the-art LLMs.

Commercial APIs & Machine Learning: We include commercial content moderation services, namely Perspective API and Tencent API [49]. Additionally, traditional machine learning algorithms, including Support Vector Machine (SVM), Random Forest (RF), k-Nearest Neighbors (k-NN), and AdaBoost, are utilized as baselines relying on handcrafted features.

Deep Learning Models: For standard neural network architectures, we employ TextFNN [50] and TextCNN [51] for basic text embedding and local n-gram feature extraction. To capture long-range contextual dependencies and sequential patterns, we compare against RNN-based variants: Bi-LSTM [52], BiLSTM-Att (with attention mechanism) [53], BiLSTM-CRF [54], and RCNN [55], which optimally integrates CNNs and RNNs.

PLMs: We utilize prominent Transformer-based models, including BERT [56] and its robustly optimized variant RoBERTa, which excel in context-aware representation learning. We also incorporate DistilBERT, a lightweight, knowledge-distilled variant of BERT designed for fast inference, and DeBERTa, which employs disentangled attention mechanisms to capture fine-grained semantic dependencies. Furthermore, we include SBERT, an efficient Siamese network-based extension for generating high-quality fixed-length sentence embeddings.

LLMs: Finally, to benchmark against contemporary generative models, we select a wide range of LLMs. This includes advanced proprietary models such as GPT-4o, GPT-4-Turbo, and the reasoning-focused OpenAI-o1. Furthermore, we evaluate powerful open-weight bilingual models, encompassing LLaMA3-70B, ChatGLM3-6B, Qwen2-72B, DeepSeek-V3, and DeepSeek-R1, to assess their zero-shot and reasoning capabilities in complex semantic tasks.

#### 4.1.3. Evaluation Metrics

To comprehensively evaluate model performance, this experiment selects Accuracy, Precision, Recall, F1 score, Pearson Correlation (PC), and Spearman’s Rank Correlation (SRC) as evaluation metrics.

Among these, the Pearson Correlation and Spearman’s Rank Correlation are used to measure the linear correlation between two variables, specifically analyzing the linear association between model representations and specific labels, and evaluating the consistency with human judgments, respectively.

#### 4.1.4. Evaluation Settings for LLMs and APIs

To ensure a fair and rigorous comparison between the proposed HED-MSP and baseline methods, we standardize the evaluation conditions across all models. Specifically, all models are evaluated on the same HECD test set using identical input text, without any additional preprocessing or data augmentation.

For traditional machine learning methods and neural classifiers, all models are fully fine-tuned on the training split of the HECD dataset.

For commercial APIs (Perspective API and Tencent API), we query their official endpoints using the same test instances and adopt their default confidence thresholds for binary classification.

For LLMs, to ensure a fair comparison and avoid the instability commonly observed in naive zero-shot settings, we evaluate all LLMs (including GPT-4o, DeepSeek series, and Qwen series) using a unified task-specific prompt template. The system prompt defines the model’s role and provides a clear definition of harmful euphemisms, along with structured output constraints to ensure consistent classification decisions.

To improve reproducibility and reduce randomness, the generation temperature is set to a low value (T=0.05), ensuring stable and near-deterministic outputs across runs.

#### 4.1.5. Experimental Setup

All experiments were implemented using the PyTorch framework and conducted on a server equipped with dual NVIDIA Tesla V100 GPUs (32 GB VRAM). The HED-MSP model was optimized using the AdamW optimizer with an initial learning rate of $1\times 10^{-5}$. To strike a balance between computational efficiency and GPU memory constraints, the batch size was set to 64. To mitigate overfitting, a dropout rate of 0.5 was applied. The maximum number of training epochs was set to 10, and an early stopping strategy was implemented during the training phase based on the validation set performance. Furthermore, a 10-fold cross-validation strategy was employed to robustly train and evaluate the model. To minimize experimental variance, all experiments were repeated 5 times using different random seeds, with the averaged results reported. For the baseline models, foundational encoders were initialized with their respective publicly available pre-trained weights prior to task-specific fine-tuning, and the best-performing models and hyper-parameters were selected based on the validation set to ensure a fair comparison.

## 5. Results and Analysis

### 5.1. Representation Learning Comparative Experiments

**Figure 3:**
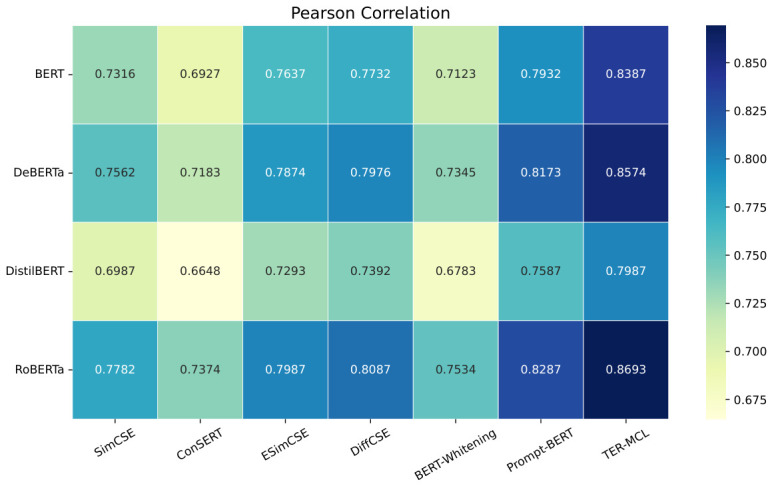
Pearson Correlation Coefficient Results for Representation Learning Comparative Experiments.

**Figure 4:**
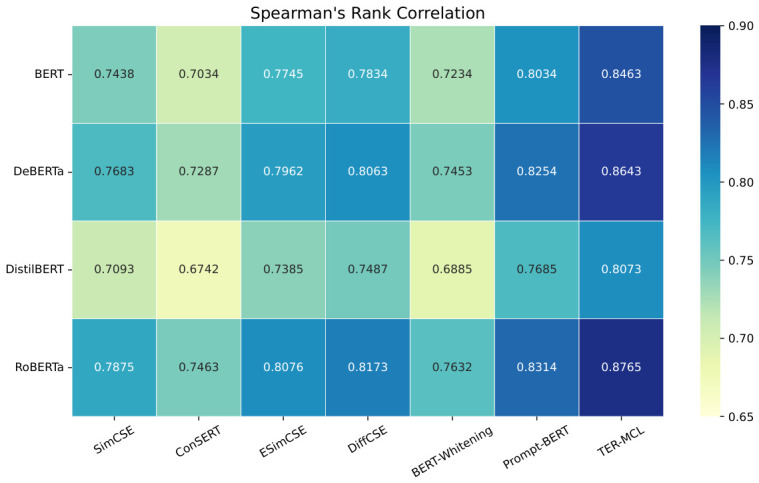
Spearman’s Rank Correlation Coefficient Results for Representation Learning Comparative Experiments.

In this experiment, comparative representation learning experiments for the HER-MCL framework were conducted on the HECD dataset. The Pearson Correlation and Spearman’s Rank Correlation coefficients are used to calculate the distribution correlation between the sentence pair representation vector similarity and the label similarity. A higher distribution correlation indicates higher representation accuracy, signifying a better representation training method. Conversely, a lower correlation indicates poorer representation accuracy and a less effective training method. The experimental results are shown in Figure 3 and Figure 4.

As shown in the figure, among all the compared methods, HER-MCL consistently achieves higher Pearson and Spearman correlation coefficients, indicating its superiority in learning semantically meaningful representations for harmful euphemism detection.

Specifically, on the DeBERTa backbone network, HER-MCL improves the Pearson correlation coefficient (PC) and Spearman rank correlation coefficient (SRC) compared to the RoBERTa-based Prompt-BERT baseline. These improvements demonstrate that the proposed framework significantly enhances the semantic consistency between representation vectors and annotation labels, particularly in capturing implicit euphemisms. Furthermore, HER-MCL also achieves consistent performance improvements on the lightweight DistilBERT model, further validating the robustness of the proposed method across different backbone network architectures. This indicates that the performance improvement is not dependent on model size but stems from the effectiveness of the proposed representation learning strategy.

Overall, the experimental results demonstrate that HER-MCL effectively improves representation quality while maintaining excellent performance on both large-scale and lightweight backbone models, making it suitable for practical deployment scenarios with stringent efficiency requirements.

### 5.2. Ablation Study of the Representation Learning Module

To evaluate the contribution of each module to the enhancement of harmful euphemism representations, we conducted an ablation study on the deep prompt chain-of-thought (DPTC), data augmentation, and multi-head contrastive learning (MCL) components of the HER-MCL framework using the HEC-STS dataset. The specific configurations for the ablated models are as follows:

- HER-MCL (-DPTC): Removes the deep prompt chain-of-thought module, employing a vanilla contrastive learning mechanism.
- HER-MCL (-mapping): Removes the mapping-based augmented samples from the three-fold data augmentation module, utilizing only the other two types to construct contrastive learning samples.
- HER-MCL (-replacing): Removes the replacement-based augmented samples from the three-fold data augmentation module, utilizing only the other two types to construct contrastive learning samples.
- HER-MCL (-MCL): Removes the multi-head contrastive learning mechanism, relying solely on the SimCSE mechanism.

As shown in Table 5, the complete HER-MCL framework consistently achieves the best performance across all backbone models, demonstrating the effectiveness of jointly integrating DPTC, multi-strategy data augmentation, and multi-head contrastive learning. The results indicate that harmful euphemism representation learning benefits from both external knowledge enhancement and structured representation optimization.

When the DPTC module is removed, the performance drops significantly across all models, suggesting that the structured reasoning process and external knowledge provided by DPTC play a crucial role in enriching semantic representations. Without this module, the model relies solely on surface-level textual information, making it more difficult to capture the implicit and context-dependent nature of harmful euphemisms.

Ablation results on data augmentation strategies further reveal differentiated and complementary contributions. Removing mapping-based augmentation leads to a substantial performance decrease, indicating that linking euphemistic expressions with their underlying harmful meanings is essential for semantic alignment. In contrast, removing replacement-based augmentation results in a relatively smaller but consistent degradation, suggesting that this strategy primarily enhances structural robustness by introducing lexical and contextual variations, allowing the model to learn invariant semantic features under diverse surface forms. Notably, randomized augmentation is treated as a fundamental stochastic regularization baseline (e.g., word dropout and shuffling) rather than an explicit semantic transformation. Its contribution to representation stability and overfitting mitigation is implicitly reflected in the overall system performance; therefore, the ablation study focuses on augmentation strategies that provide task-specific semantic guidance.

In addition, the removal of the multi-head contrastive learning (MCL) mechanism results in one of the most significant performance declines, highlighting its critical role in representation learning. Compared to standard contrastive learning, the multi-head design enables the model to learn complementary semantic structures in multiple subspaces, thereby improving its ability to capture fine-grained semantic relationships.

### 5.3. Model Comparison Experiment

We tested the proposed HED-MSP model and the baselines on the HECD dataset; the experimental results are shown in Table 6.

This performance demonstrates that HED-MSP achieves a substantial improvement over all categories of baseline methods, including API-based systems, traditional machine learning models, deep neural classifiers, and large language models.

Among API-based systems such as Perspective-API and Tencent-API, we observe limited capability in capturing implicit harmful intent. These methods tend to exhibit relatively high precision but low recall, indicating that rule-based or externally pretrained moderation systems are insufficient for identifying euphemistic expressions with indirect or context-dependent semantics. For traditional machine learning methods, models such as RF and k-NN achieve moderate performance; however, their reliance on shallow lexical and statistical features restricts their ability to generalize to evolving euphemistic patterns. This limitation becomes more pronounced when dealing with semantically dynamic or culturally embedded expressions. Neural network-based toxic content classifiers further improve performance, with RoBERTa-base achieving the strongest baseline F1-score. Nevertheless, these models still struggle to fully capture implicit semantic shifts in euphemistic language, particularly when rhetorical or contextual transformations are involved. Large language models, including GPT-4o and DeepSeek-series models, demonstrate strong semantic understanding and relatively high recall. However, they consistently suffer from lower precision, as they tend to over-interpret semantically ambiguous euphemistic expressions and assign toxic labels to benign or context-dependent content. This limitation arises from their unconstrained generative inference paradigm, which lacks explicit structural constraints during semantic reasoning.

In contrast, HED-MSP achieves a better balance between precision and recall. We attribute this improvement to the proposed Syntactic Semantic Perception Module (SSPM), which introduces explicit syntactic constraints into the semantic reasoning process. By leveraging dependency-aware structural modeling, SSPM imposes hierarchical boundaries on representation learning, thereby reducing spurious semantic associations and mitigating misclassification in neutral or ambiguous contexts. Furthermore, the empirical results suggest that the proposed multi-dimensional semantic perception fusion framework is capable of capturing subtle harmful intentions that are often overlooked by surface-level token-based analysis. By integrating syntactic, contextual, and rhetorical semantic signals, HED-MSP learns more robust and discriminative representations, enabling more effective identification of evolving euphemistic expressions in dynamic content moderation scenarios.

Overall, HED-MSP outperforms all baselines, achieving a 93.23% F1 score. The improvement over the strongest non-LLM baseline (RoBERTa-base) demonstrates the effectiveness of multi-dimensional semantic perception fusion. Moreover, compared to LLM-based methods, HED-MSP achieves not only higher accuracy but also significantly lower inference cost, making it more suitable for real-world high-throughput content moderation systems.

### 5.4. Real-Time Performance Experiments

The HED-MSP model is designed as a lightweight architecture for harmful euphemism detection under real-time moderation constraints. In such scenarios, low latency and high throughput are critical for handling high-concurrency online requests. To evaluate the deployment feasibility of HED-MSP, we conduct a systematic analysis of its inference efficiency and computational cost.

To comprehensively evaluate the models, we define the following metrics: (1) Latency (ms/sample): The average inference time for a single input sample without batching; (2) Throughput (samples/s): The number of samples processed per second under batched or concurrent inference settings; (3) Memory Usage (GB): Peak GPU memory consumption during inference. (4) Model Parameters (B): The number of model parameters; (5) Inference Cost (Yuan/1M tokens): The estimated monetary cost per million tokens.

It is important to emphasize that LLMs are used exclusively in the offline stage for generating chain-of-thought supervision signals. Therefore, all reported metrics for HED-MSP reflect only its online inference efficiency.

As illustrated in Table 7, the HED-MSP method achieves an effective balance between inference efficiency and computational cost. Compared to strong encoder-based baselines such as BERT and RoBERTa, it provides lower latency and higher throughput with a reduced memory footprint. Compared to generative LLMs, HED-MSP offers orders-of-magnitude improvements in response time and deployment cost. While massive LLMs exhibit strong zero-shot generalization ability, their inference is significantly constrained by high computational overhead and latency. By leveraging LLMs strictly in the offline stage, our framework retains their reasoning capability without sacrificing the millisecond-level online efficiency required for high-concurrency moderation tasks.

### 5.5. Ablation Study

To evaluate the detection performance improvements brought by the proposed Syntactic Semantic Perception Module (SSPM), Contextual Semantic Perception Module (CSPM), Rhetorical Semantic Perception Module (RSPM), and Cross-channel Dynamic Adaptive Fusion (CDAF) module for harmful euphemisms, this experiment conducts an ablation study on the modules of the HED-MSP framework to demonstrate the contribution of each component and mechanism to the task. The comparative ablation models are set as follows:

- HED-MSP (-SSPM): Removes the syntactic lexical semantic perception module, retaining the rest.
- HED-MSP (-CSPM): Removes the contextual background semantic perception module, retaining the rest.
- HED-MSP (-RSPM): Removes the rhetorical behavior semantic perception module, retaining the rest.
- HED-MSP (-CDAF): Removes the cross-channel fusion module, retaining the rest.

As shown in Table 8, the complete HED-MSP framework achieves the best overall performance, reaching an F1 score of 93.23%. This result significantly outperforms the representative backbone model RoBERTa-base and is also competitive with advanced large language model baselines such as OpenAI-o1. These empirical findings indicate that relying solely on general-purpose contextual encoders or prompt-based LLM inference is insufficient for effectively disentangling highly complex euphemistic expressions, thereby clearly demonstrating the necessity of the proposed multi-dimensional semantic perception architecture.

Furthermore, the ablation results show that removing any individual module leads to a noticeable performance degradation, although the extent of the decline varies depending on the specific cognitive role of each component. This observation suggests that there exist complex and complementary interactions among different modules in the task of harmful euphemism detection.

Specifically, removing the rhetorical semantic perception module (−RSPM) results in the most significant performance drop. The RSPM is designed to decode non-literal expressions, particularly rhetorical devices such as metaphor, sarcasm, and presupposition. Since rhetorical camouflage is one of the primary mechanisms used in harmful euphemisms, removing this module significantly reduces the model’s sensitivity to implicit malicious intent, making it more vulnerable to being misled by surface-level benign expressions.

The second largest degradation is observed when the contextual semantic perception module (−CSPM) is removed. The CSPM focuses on modeling contextual coherence through a context-aware pooling mechanism, capturing both local and global semantic dependencies. In real-world social media scenarios, isolated sentences may appear neutral or even positive; only when considered within surrounding contextual information can their underlying harmful intent be correctly identified. Without CSPM, the model suffers from fragmented contextual understanding, leading to a substantial drop in recall.

In addition, removing the syntactic semantic perception module (−SSPM) leads to a 1.78% decrease in F1 score. The SSPM captures syntactic structure and lexical-semantic interactions, providing fundamental linguistic constraints for detection. For instance, in sentences involving negation structures, SSPM can accurately identify the scope of negation, preventing neutral statements from being misclassified as offensive content. The absence of SSPM weakens syntactic disambiguation capability and consequently degrades detection accuracy.

Finally, replacing the cross-channel dynamic adaptive fusion (CDAF) mechanism with simple feature concatenation results in a 1.05% drop in F1 score. This highlights that naive feature fusion cannot effectively adaptively prioritize the most informative semantic cues across heterogeneous representations. In contrast, the proposed dynamic fusion mechanism plays a crucial role in integrating multi-dimensional semantic signals, thereby maximizing the overall discriminative power of the model.

### 5.6. Model Generalization Experiments

We evaluate the generalization capability of HED-MSP under zero-shot conditions using different topic types from the HECD dataset.

In this experiment, all models are trained on the “General Insults” category of the HECD dataset and evaluated on the “Group Attacks” category (and vice versa). The experimental results are shown in Table 9.

As shown, HED-MSP demonstrates competitive cross-topic generalization performance. Compared to traditional lightweight classifiers, HED-MSP achieves significant performance gains across both transfer settings. For instance, in the “Group Attacks → General Insults” setting, HED-MSP’s accuracy surpasses RoBERTa-large by over 15 percentage points. Similar gains are observed in the reverse transfer scenario, indicating that our model possesses superior cross-topic adaptability among specialized, compact architectures.Simultaneously, we observe that advanced LLMs, such as GPT-4o and DeepSeek-R1, achieve superior performance in zero-shot transfer tasks. This underscores the inherent advantage of massive pre-training when encountering entirely unseen topics. Consequently, while HED-MSP does not exceed the performance ceiling set by the strongest LLM baselines, it maintains a highly competitive transfer capability with a relatively narrow performance gap.From a practical deployment perspective, HED-MSP strikes an optimal balance between generalizability and operational efficiency. Compared to massive LLMs, HED-MSP drastically reduces computational overhead and supports faster inference speeds, which is essential for large-scale, real-time content moderation. In summary, these results demonstrate that HED-MSP’s core strength lies in its ability to reconcile robust transfer performance with high real-world deployability.

### 5.7. Cross-Dataset Experiments

To evaluate the robustness of HED-MSP across diverse linguistic environments, we conducted cross-dataset experiments on three English euphemism datasets: EACL [57], FigLang [58], and JointEDI [59]. These datasets vary significantly from the Chinese-based HECD in terms of domain and annotation style, providing a rigorous testbed for assessing whether the learned detection capabilities can generalize beyond the original linguistic context.

The rationale for this cross-lingual transfer is rooted in the observation that harmful euphemisms across different languages often exhibit congruent high-level semantic and pragmatic characteristics. Specifically, such expressions rely on universal mechanisms like indirect referentiality and intent-driven semantic shifts rather than language-specific lexical markers. Our objective is to investigate whether HED-MSP can capture these language-agnostic linguistic structures. The experimental results are shown in Table 10.

As shown, HED-MSP consistently outperforms all lightweight baselines across the three English datasets. Compared to traditional neural architectures like BiLSTM and RoBERTa variants, HED-MSP achieves a significant performance uplift. This demonstrates that our framework, through Deep Prompt CoT and multi-dimensional semantic modeling, effectively captures generalizable patterns that transcend surface-level linguistic features.

While advanced LLMs like GPT-4o and DeepSeek-R1 demonstrate superior performance in certain cross-lingual settings—reflecting the advantages of massive multilingual pre-training—HED-MSP maintains highly competitive performance. Most importantly, it achieves this while offering substantially lower computational overhead and inference latency, reconciling robust cross-dataset generalizability with the efficiency required for real-world deployment.

### 5.8. Case Study

While the HED-MSP framework exhibits robust overall performance, analyzing its failure cases and underlying error patterns is essential for guiding future enhancements. We conducted a manual error analysis on several false negative (FN) and false positive (FP) samples, as detailed in Table 11.

As observed, the model misclassified harmful euphemisms such as “animals” and “stick” as harmless. Regarding Case 1, the primary reason is that our methodology relies heavily on textual context. In isolation, the comment appears semantically neutral; however, its toxicity as a racial slur is “activated” only when posted under videos featuring specific minority groups. Similarly, in Case 2, the harmfulness of the euphemism is largely defined by the accompanying non-textual emoticons. These instances reveal a “physical boundary” of the current model, namely the lack of capability to integrate non-textual information such as video frames and visual emojis, allowing such extreme cross-modal implicit toxicity to evade detection.

Conversely, Cases 3 and 4 illustrate false positive scenarios. In Case 3, a specific community utilizes “China aluminum” (Guolü) within a sarcastic structure as a form of humorous self-deprecation rather than an actual attack. From the perspective of detection logic, the inclusion of rhetorical sarcasm may cause the RSPM module to mislead the fusion mechanism into a conservative classification of offensive speech. This suggests that while our approach can detect rhetorical anomalies, it suffers from a “knowledge lag” concerning emerging subcultures. Furthermore, in Case 4, HED-MSP misclassified a harmless comment refuting the term “Woman’s Fist” as a harmful euphemism. For such comments, despite the presence of sensitive terms, the macro-semantic intent—characterized by explicit quotation and refutation—is typically benign. This indicates a limitation in our framework’s ability to comprehend high-level pragmatic intentions.

In future research, we aim to integrate multi-modal perception to process non-textual information like video and emojis. Additionally, we plan to develop dynamic pragmatic reasoning mechanisms to effectively distinguish genuine malicious attacks from harmless quotations or subcultural self-mockery.

## 6. Conclusions

This paper presents HED-MSP, a multi-dimensional semantic-aware framework for harmful euphemism detection. Unlike prior methods that primarily rely on surface-level lexical matching or single-granularity contextual representations, HED-MSP models euphemistic expressions from three complementary perspectives, namely syntactic structure, contextual semantics, and rhetorical behavior, and integrates them through a cross-channel dynamic adaptive fusion mechanism. Experimental results demonstrate that the proposed framework consistently achieves state-of-the-art performance among specialized lightweight architectures. Moreover, it exhibits strong robustness and generalization ability across different backbone encoders, striking an optimal balance between competitive transferability and real-time operational efficiency when compared to massive LLMs.

The contributions of this work are twofold, including both dataset construction and methodological advances in representation learning. First, we construct the HECD dataset, which provides fine-grained annotations of harmful euphemisms along with topic categories, attack types, and semantic explanations, thereby addressing the lack of structured resources in this research area and supporting fine-grained semantic learning.

Second, from a methodological perspective, we propose a multi-dimensional semantic modeling framework with two key innovations. On the one hand, we demonstrate that cognitive reasoning signals encoded by large language models (LLMs) through Chain-of-Thought (CoT) prompting can be effectively transferred to lightweight encoders via offline knowledge-enhanced contrastive learning. This design enables the incorporation of external reasoning capabilities into compact models while avoiding the computational overhead of direct LLM inference. On the other hand, we show that effective euphemism understanding requires unified representation learning that jointly captures syntactic dependency relations, contextual coherence, and rhetorical transformation patterns. This multi-granularity modeling paradigm provides a more structured and interpretable way to represent implicit harmful semantics.

Beyond performance improvements, HED-MSP also provides practical implications for real-world deployment. By decoupling computationally expensive representation enhancement from online inference, the proposed framework enables efficient deployment in large-scale and low-latency content moderation systems. Moreover, since the model focuses on language-agnostic semantic structures rather than surface lexical cues, it exhibits strong potential for multilingual and cross-domain extension.

In future work, we will further investigate robust learning strategies under noisy and imbalanced data distributions, and explore cross-lingual adaptation mechanisms to improve generalization across diverse linguistic environments. In addition, we plan to incorporate external knowledge resources and investigate model compression techniques to further enhance scalability and efficiency for real-world high-throughput moderation scenarios.

## Figures and Tables

**Table 1:** Comprehensive Comparison of State-of-the-Art Harmful Euphemism Detection Methods.

Category	Reference	Specific Dataset	Backbone Model	External Knowledge	
				Lexicon	LLM/CoT
Rules & Static Rep.	Davidson [5]	Twitter tweets	LR, SVM, Random Forest	√	×
	Waseem [6]	Twitter tweets	LR, SVM	√	×
	Yuan [7]	Dark Web	Word2Vec	√	×
	Taylor [8]	DailyStormer, Twitter	Word2Vec + PageRank	√	×
Dynamic Rep.	Zhu [9]	Reddit, Gab	BERT	×	×
	Huang [10]	Tieba, Sina Weibo	Transformer	×	×
	Guan [11]	Online Communities	RoBERTa	×	×
Contrastive Learning	Kim [12]	IHC, SBIC, DynaHate	Dual-Encoder (BERT)	×	×
	Lu [13]	OffensEval, HatEval	Dual-Encoder (RoBERTa)	×	×
	Deng [14]	AugCOLD	Multi-teacher Distillation	×	×
	Hartvigsen [15]	ToxiGen	HateBERT, RoBERTa	×	×
Structure & Multimodal	Pavlopoulos [16]	TOXICSPANS	Sequence Labeling	×	×
	Wang [17]	CBKD	Feature Adapter BERT	√	×
	Huang [18]	Weibo	GCN	×	×
	Ghosh [19]	Reddit	Hyperbolic Network	×	×
	Zhang [20]	MultiOFF, Memotion	Optimal Transport Framework	×	×
LLM Reasoning	Wei [21]	GSM8K, SVAMP	PaLM, GPT-3 (CoT)	×	√
	Kojima [22]	MultiArith	GPT-3 (Zero-shot)	×	√
	Zhao [23]	CSQA, OpenBookQA	LLM + GNN	×	√
	Mondal [24]	ScienceQA	LLM + Vision + KG	×	√

Note: √ in “Lexicon” indicates reliance on curated sensitive vocabularies or phonetic/glyph rules; √ in “LLM/CoT” denotes utilizing LLMs for generation or reasoning. × indicates that the corresponding feature or mechanism is not utilized. Literature under “LLM Reasoning Paradigms” represents foundational semantic reasoning frameworks in general NLP tasks, serving as our methodological basis.

**Table 2:** The initial keyword list for the toxic euphemism comment crawler.

Topic Category	Seed Keyword List (Examples in Chinese)
Group Attacks	猩猩 [Lit: “Gorilla”, a racist slur for people of African descent], 幕刃 [Lit: “Twilight Blade”, a homophonic derogatory term for women].
Vulgar Speech	金针菇 [Lit: “Enoki Mushroom”, denoting male genitalia], 菊花 [Lit: “Chrysanthemum”, denoting the anus].
Political Content	大毛 [Lit: “Big Fur”, implying Russians], 棒子 [Lit: “Stick”, a derogatory slur for Koreans].
General Insults	鸡 [Lit: “Chicken”, a metaphor for sex workers], 孙子 [Lit: “Grandson”, an insult to one’s lineage].

Note: Given the highly offensive, vulgar, and politically sensitive nature of certain terms, and in strict compliance with standard academic ethical guidelines for toxic language research, we have redacted the most extreme raw vocabulary from the manuscript. We present only representative examples categorized by semantic domain.

**Table 3:** Statistics of the HECD Dataset.

Topic Type	Attack Type								Harmful	Harmless
	HP	PS	VS	Meme	Abbrev.	MP	Irony	CP		
Group Attacks	356	370	514	586	499	633	771	589	4318	5594
Vulgar Speech	496	412	294	123	599	480	322	331	3057	3356
Political Content	146	235	128	164	217	589	288	186	1953	2913
General Insults	230	188	94	11	298	416	499	417	2153	3173
Total	1228	1205	1030	884	1613	2118	1880	1523	11,481	15,036

Note: In this table, HP denotes homophone, PS denotes polysemy, VS denotes visual similarity, MP denotes metaphor, CP denotes compound.

**Table 4:** Deep Prompt CoT Template Across Reasoning Stages.

Stage & Objective	Deep Prompt CoT Template
Phase I: Task Definition Obj: Understand requirements and task formulation.	At this stage, I assume the role of a linguistics and cognitive science expert with specialized knowledge in harmful euphemism analysis. The input consists of: (i) a target sample or text segment containing potential harmful euphemisms, (ii) identified euphemistic expressions, (iii) corresponding semantic interpretation labels, and (iv) auxiliary prompt seed words. The expected output includes: (i) cognitive reasoning analysis of the input sample, (ii) semantically similar samples, (iii) extracted euphemistic expressions, (iv) mapped harmful semantic interpretations, and (v) refined cognitive reasoning chains. A step-by-step reasoning process is required, integrating all relevant linguistic and contextual factors to produce a structured analytical output.
Phase II: Cognitive Understanding Obj: Formulate structured understanding of euphemisms.	In this stage, I establish a formal understanding of harmful euphemisms. From a semantic perspective, harmful euphemisms refer to textual expressions in which discriminatory, violent, or illegal intents are concealed through euphemistic transformations. From a structural perspective, they may appear as either short phrases or long-form discourse units, while euphemistic expressions typically exist at lexical or phrasal levels with context-dependent meanings. Furthermore, harmful euphemism interpretation is guided by five core cognitive dimensions, including eight types of attack strategies: homophony, polysemy, visual similarity, meme-based transformation, abbreviation, metaphor, irony, and compound structures.
Phase III: Prompt Alignment Obj: Analyze semantic alignment with examples.	Given the current input sample and provided prompt examples, I analyze their semantic relationships and describe how both jointly contribute to fulfilling the task objective. In particular, I evaluate semantic similarity between the target sample and reference examples, and identify key divergences in linguistic expression and intent encoding.
Phase IV: Cognitive Reasoning Obj: Derive structured reasoning chains.	I conduct a detailed analysis of how harmful euphemistic expressions are manifested within the input text and how they are interpreted as implicit harmful meanings. Based on the defined cognitive framework, I identify specific attack strategies and construct a structured reasoning chain explaining the detection process. This includes linguistic interpretation grounded in cognitive linguistics principles and logical inference over euphemistic transformations.
Phase V: Target Generation Obj: Generate new samples with semantic mappings.	Based on the task description and cognitive analysis, I generate semantically similar harmful euphemistic expressions. I further identify corresponding euphemistic terms and their mapped harmful meanings. A structured representation is required in the form: euphemistic term–attack strategy–semantic interpretation. Additionally, generated samples must be consistent with reference examples and cognitive reasoning constraints.
Phase VI: Output Evaluation Obj: Ensure quality and task compliance.	Finally, I evaluate whether the generated outputs satisfy all task constraints. This includes assessing semantic correctness, structural consistency, and alignment with prior definitions. If the outputs do not meet the requirements, the process returns to Stage 2 for iterative refinement. Otherwise, the final results are accepted and output.

**Table 5:** The results of the ablation experiment for HER-MCL.

Method	Pre-Trained Model	Pearson Correlation	Spearman’s Rank Correlation
HER-MCL (-DPTC)	BERT	0.7924	0.7948
	DeBERTa	0.7543	0.7689
	DistilBERT	0.7256	0.7327
	RoBERTa	0.7823	0.7839
HER-MCL (-mapping)	BERT	0.8067	0.8135
	DeBERTa	0.7629	0.7812
	DistilBERT	0.7359	0.7416
	RoBERTa	0.8114	0.8032
HER-MCL (-replacing)	BERT	0.8239	0.8297
	DeBERTa	0.7784	0.7928
	DistilBERT	0.7523	0.7584
	RoBERTa	0.8337	0.8219
HER-MCL (-MCL)	BERT	0.7532	0.7536
	DeBERTa	0.7689	0.7886
	DistilBERT	0.7187	0.7353
	RoBERTa	0.7976	0.7951
HER-MCL (Ours)	BERT	0.8387	0.8463
	DeBERTa	0.8574	0.8643
	DistilBERT	0.7987	0.8073
	RoBERTa	0.8693	0.8765

*Note:* In this table, DPTC denotes deep prompt chain-of-thought, mapping denotes mapping-based augmented samples, replacing denotes replacement-based augmented samples, and MCL denotes multi-head contrastive learning.

**Table 6:** Performance comparison of HED-MSP with baseline methods.

Category	Model	Acc. (%)	Pre. (%)	Rec. (%)	F1 (%)
API-based Systems	Perspective-API	61.33	85.89	16.75	28.04
	Tencent-API	62.64	66.67	33.84	44.89
Machine Learning Methods	RF	81.42	79.57	78.97	79.27
	SVM	69.98	66.63	66.67	66.65
	k-NN	75.49	69.65	80.67	74.76
	AdaBoost	67.58	64.15	63.33	63.74
Toxic Content Classifiers	TextFNN	84.01	84.07	81.04	82.53
	TextCNN	73.10	71.23	67.39	69.25
	RCNN	83.20	82.50	80.10	81.25
	BiLSTM	87.96	87.88	85.67	86.76
	BiLSTM-Att	88.19	88.35	85.79	87.05
	BiLSTM-CRF	88.30	88.20	86.50	87.30
	BERT-base	88.81	86.69	88.99	87.60
	BERT-large	88.37	81.36	92.06	86.14
	RoBERTa-base	89.97	87.56	90.61	88.90
	RoBERTa-large	89.01	85.66	90.67	87.95
	SBERT	86.53	84.43	85.43	84.92
Large Language Models	GPT-4o	72.54	63.06	93.97	75.48
	GPT-4-Turbo	77.26	70.67	84.48	76.96
	OpenAI-o1	85.35	85.12	85.48	85.30
	Qwen2-72B	70.17	61.86	87.82	72.59
	LLaMA3-70B	55.13	51.45	4.16	7.69
	ChatGLM3-6B	56.52	64.32	7.49	13.42
	DeepSeek-V3	83.64	83.27	82.92	83.15
	DeepSeek-R1	84.85	84.63	85.12	84.88
HED-MSP (Ours)		93.94	93.09	93.36	93.23

**Table 7:** Comprehensive comparison of real-time efficiency and computational cost.

Model	Latency	Throughput	Memory	Params	Cost
	(ms/Sample)	(Samples/s)	(GB)	(B)	(Yuan/1M)
BERT-large	5–10	150–250	8–10	0.34	∼0
RoBERTa-large	5–10	150–250	8–10	0.25	∼0
ChatGLM3-6B	50–100	40–80	15–20	6.0	∼0
LLaMA3-70B	1000–2000	5–15	70–80	70.0	∼0
Qwen2-72B	1000–2000	5–15	60–80	72.0	3–5
DeepSeek-V3	1500–3000	20–50	N/A	N/A	2–8
DeepSeek-R1	2000–4000	20–50	N/A	N/A	4–16
GPT-4o (API)	1000–2500	50–100	N/A	N/A	35–105
GPT-4-Turbo	1500–3000	∼50	N/A	N/A	70–210
HED-MSP (Ours)	2–5	300–500	4–9	0.23	∼0

Note: “N/A” indicates not applicable, as commercial API models are accessed remotely, making local memory and parameter counts unavailable. Latency and throughput for API-based models are measured under concurrent request settings. Throughput may vary depending on batching and service-side rate limits, and is therefore reported as a range or approximation.

**Table 8:** Results of the Ablation Study with Baseline References.

Model	Accuracy (%)	Precision (%)	Recall (%)	F1 (%)
RoBERTa-base	89.97	87.56	90.61	88.90
OpenAI-o1	85.35	85.12	85.48	85.30
HED-MSP (-SSPM)	92.15	91.32	91.58	91.45
HED-MSP (-CSPM)	91.73	90.85	91.12	90.98
HED-MSP (-RSPM)	91.35	90.47	90.74	90.60
HED-MSP (-CDAF)	92.84	92.05	92.32	92.18
HED-MSP (Ours)	93.94	93.09	93.36	93.23

**Table 9:** Results of Generalization Experiments.

Baseline Type	Baseline Name	Group Attacks → General Insults			General Insults → Group Attacks		
		Acc. (%)	Pre. (%)	F1 (%)	Acc. (%)	Pre. (%)	F1 (%)
Harmful Content Classifier	TextFNN	56.85	53.91	52.50	55.28	55.20	52.08
	TextCNN	55.14	52.37	50.88	53.57	53.46	50.56
	RCNN	55.50	53.31	51.56	54.33	52.70	49.49
	BiLSTM	56.93	54.05	56.02	54.46	51.10	47.13
	BiLSTM-Att	55.76	53.57	53.59	53.97	50.92	48.85
	BiLSTM-CRF	57.83	55.52	54.27	56.33	53.54	52.81
	BERT-base	61.85	58.61	59.06	60.06	55.80	57.68
	BERT-large	62.89	60.18	57.43	57.21	53.45	53.64
	RoBERTa-base	60.27	59.29	52.89	57.11	53.90	54.91
	RoBERTa-large	64.63	62.64	59.27	58.43	54.97	55.10
	SBERT	50.73	48.37	40.72	53.76	51.06	54.75
LLMs	GPT-4o	84.05	76.62	86.13	72.00	65.44	77.02
	GPT-4-Turbo	83.20	75.04	84.54	70.51	63.84	75.67
	Qwen2-72B	80.17	73.55	82.91	68.95	63.99	73.80
	LLaMa3-70B	47.84	41.67	7.58	49.90	51.43	12.00
	ChatGLM3-6B	46.55	14.29	1.57	51.62	63.16	15.84
	DeepSeek-V3	68.50	65.00	66.80	62.00	56.30	60.50
	DeepSeek-R1	79.20	72.50	81.00	69.80	64.20	72.50
Our Model	HED-MSP	79.96	75.24	78.43	66.05	63.16	65.55

**Table 10:** Performance comparison on EACL, FigLang, and JointEDI datasets.

Baseline Type	Model	EACL			FigLang			JointEDI		
		Pre.	Rec.	F1	Pre.	Rec.	F1	Pre.	Rec.	F1
Harmful Content Classifier	TextCNN	80.85	85.31	82.99	71.95	77.73	74.69	86.74	89.90	88.28
	BiLSTM	80.86	83.42	82.12	65.04	64.75	64.86	83.23	85.26	84.21
	BiLSTM-Att	80.33	80.45	80.20	72.58	72.87	72.71	86.40	86.51	86.45
	BiLSTM-CRF	81.30	82.05	81.64	73.89	71.78	72.74	85.54	87.17	86.31
	RCNN	80.86	83.42	82.12	72.58	72.87	72.71	86.40	86.51	86.45
	BERT-base	83.91	88.15	85.93	72.71	81.83	76.86	86.48	90.87	88.60
	BERT-large	84.12	86.75	85.39	78.69	80.38	79.38	87.54	87.78	87.63
	RoBERTa-base	81.45	90.99	85.85	70.14	87.08	77.58	85.82	83.69	84.71
	RoBERTa-large	82.18	93.56	87.47	72.48	82.40	76.80	85.11	88.44	86.66
LLMs	LLaMa3-70B	77.42	73.97	75.66	66.88	82.24	73.77	79.92	50.76	62.09
	ChatGLM3-6B	71.56	50.00	58.87	67.34	72.13	69.66	90.79	68.84	78.31
	Qwen2-72B	83.75	79.45	81.55	68.43	95.35	79.68	79.95	58.57	67.61
	DeepSeek-V3	84.50	91.00	87.50	77.85	84.20	80.89	86.30	90.75	88.44
	DeepSeek-R1	85.65	91.25	88.35	79.12	85.40	82.13	89.70	89.15	**89.42**
	GPT-4o	84.09	88.70	86.33	72.63	92.08	81.20	86.83	58.38	69.82
	GPT-4-Turbo	83.40	75.68	79.35	73.05	87.43	79.60	92.00	43.14	58.89
Our Model	HED-MSP	85.16	90.41	87.71	77.47	83.61	80.42	89.48	88.79	89.13

**Table 11:** Failure cases analysis of HED-MSP.

Euphemisms (Connotation)	Comments	True Label	Prediction
Animals (Black people) 动物（黑人）	Case 1. I can only say, Humans and animals cannot mix together. 案例1. 我只能说，人跟动物[猴子表情]就不能混在一起	Harmful	Harmless
Stick (Korean & Korea) 棒子（韩国人及韩国）	Case 2. Seriously, who actually likes those sticks? Can’t you tell your “oppas” to hit the gym a bit more? 案例2. 到底谁喜欢棒子啊，让你家哥哥多练练好吗[恶心表情*3]	Harmful	Harmless
China aluminum (Chinese females) 国铝（中国女性）	Case 3. Sorry, we domestic aluminum, who are short in height and low in education, are simply not good enough for you. 案例3. 不好意思，我们身高矮、学历低的国铝根本配不上您。	Harmless	Harmful
Woman’s Fist (Feminism) 女拳（女权）	Case 4. You cannot just say “woman’s fist” the moment you see a woman. Isn’t it better for everyone to get along peacefully? 案例4. 你们不能看到一个女的就说女拳，大家和平相处不好嘛	Harmless	Harmful

## Data Availability

To prevent the potential misuse of the harmful content and euphemism techniques detailed in the HECD dataset by malicious actors, the raw dataset is not deposited in a fully public repository. However, the fully anonymized dataset is available on request from the corresponding author for non-commercial academic purposes, subject to a data-sharing agreement ensuring ethical usage.
